# Supplementary material for: Biomedical knowledge graph-optimized prompt generation for large language models
Source: Bioinformatics. 2024 Sep 17;40(9):btae560. doi: 10.1093/bioinformatics/btae560 (PMC11441322; doi:10.1093/bioinformatics/btae560)
Supplement: btae560_Supplementary_Data [file btae560_supplementary_data.docx]

***Biomedical knowledge graph-optimized prompt generation for large language models***

Soman et al.

**Supplementary Material**

**SPOKE and its applications**

SPOKE stands for ‘Scalable Precision Medicine Open Knowledge Engine’. It serves as a comprehensive biomedical knowledge engine. It's a massive knowledge graph that integrates information from 41 different biomedical databases, connecting 42 million nodes of 28 types through 160 million edges of 91 types. SPOKE functions as a vast repository of curated biomedical knowledge, linking various concepts such as genes, proteins, drugs, compounds, and diseases [(Morris et al. 2023)](https://paperpile.com/c/4FUUnF/gNIhV).

SPOKE has been utilized in numerous biomedical applications, demonstrating its versatility and power:

a) Multiple Sclerosis prediction: SPOKE was used to recognize prodromal features of multiple sclerosis and predict diagnosis by embedding electronic health records onto the knowledge network [(Nelson, Bove, et al. 2021)](https://paperpile.com/c/4FUUnF/LJbS).

b) Drug Repurposing: SPOKE was used for the systematic integration of biomedical knowledge to prioritize drugs for repurposing [(Himmelstein et al. 2017)](https://paperpile.com/c/4FUUnF/58J4).

c) Space Medicine: SPOKE was used to uncover signs and symptoms associated with terrestrial diseases from transcriptomic data of space flown mice [(Nelson, Acuna, et al. 2021)](https://paperpile.com/c/4FUUnF/w8nG).

d) Parkinson’s Disease prediction: Soman et al. demonstrated the early detection of Parkinson's disease by enriching patient electronic health records using SPOKE [(Soman et al. 2023)](https://paperpile.com/c/4FUUnF/gqev).

e) Alzheimer's Disease prediction: SPOKE was used for Alzheimer's disease prediction and to gain sex-specific biological insights [(Tang et al. 2024)](https://paperpile.com/c/4FUUnF/zHl4).

These applications underscore SPOKE's role as a crucial source of biomedical knowledge and its potential to drive advancements in precision medicine.

**Context extraction from SPOKE**

We fetched the context associated with a disease node in SPOKE utilizing the REST-API service of SPOKE (<https://spoke.rbvi.ucsf.edu/swagger/>). We made use of the ‘*/api/v1/neighborhood/*’ endpoint of the API to fetch the neighbors associated with a disease node. These neighbors constituted the biomedical context associated with that disease node. To ensure the quality of the extracted context, we imposed the following constraints:

1. Treatment associations between Compound and Disease nodes should have clinical phase >= 3
2. All Protein nodes associated with a Disease node should be from the ‘SwissProt’ database ensuring that these Protein nodes were reviewed and underwent human curation.
3. Associations between Genes and Disease nodes should not be from a text mining source.

To convert the extracted context to English language, we utilized the predicate schema of SPOKE. SPOKE utilizes a defined schema for naming the predicates as shown below:

upperCase(predicateName)_<upperCase(firstLetter(subjectType)), lowerCase(firstLetter(predicateName)), upperCase(firstLetter(objectType))>

Examples:

Assertion: Disease associates with a Gene

Predicate: ASSOCIATES_DaG

Assertion: Disease resembles Disease

Predicate: RESEMBLES_DrD

Assertion: Compound TREATS Disease

Predicate: TREATS_CtD

This allowed us to convert the extracted context triples into English language using the following rule:

(S, P, O) → Subject lowerCase(predicateName) Object

For example:

(Disease hypertension, ASSOCIATES_DaG, Gene VHL) → `Disease hypertension associates Gene VHL`

In addition to extracting the connectivity between the disease and its neighbors, we also extracted the provenance information associated with those edges. In SPOKE, provenance of an association is given as its edge attribute. This provenance information is then appended to the respective context. In addition to provenance, edges within SPOKE also have other attributes such as evidence supporting the assertion (e.g., p-value, z-score, enrichment score, etc.). Additionally, we have implemented an option for users to incorporate this evidence information by utilizing a '-e' command line option, which accepts a boolean value during the execution of the KG-RAG script. If the boolean is set to True (by default it is set to False), the evidence information will be extracted and appended to the provenance information of the respective context.

**Hyperparameter analysis**

We analyzed the performance of KG-RAG across two sets of hyperparameters such as ‘Context volume’ and ‘Context embedding model’. Context volume defines the upper limit on the number of graph connections permitted to flow from the KG to the LLM. This hyperparameter introduced a balance between context enhancement and managing the input token space of the LLM. If there are N disease entities extracted from the prompt, each entity was granted a maximum of ‘Context Volume / N’ context. In cases where the number of context entries for a disease node exceeds this cap (as they meet the selection criteria mentioned in the 'context retrieval section'), we prioritize those associations with high similarity values to the prompt until the count aligns with this prescribed limit. Therefore, when tuning this hyperparameter, we considered a range of Context Volumes, spanning from 10 associations to 200 associations. The next hyperparameter, ‘Context embedding model’, was used for pruning the disease context from the KG. We analyzed the performance of KG-RAG using two embedding models such as: ‘all-MiniLM-L6-v2’ [(Reimers and Gurevych 2019)](https://paperpile.com/c/4FUUnF/drEg) and ‘S-PubMedBert-MS-MARCO’ [(Deka, Jurek-Loughrey, and Deepak 2022)](https://paperpile.com/c/4FUUnF/5z4Xn). Acknowledging that biomedical contexts often utilize vocabulary that could differ from general domain scenarios, we aimed to determine which model exhibited a greater proficiency in retrieving the accurate biomedical context from the KG to respond to the input prompt.

**Disease entity recognition - Retrieval analysis**

We evaluated the effectiveness of the disease entity recognition module of KG-RAG framework by assessing the retrieval accuracy of disease entity extraction and subsequent matching to SPOKE disease nodes. To accomplish this, we constructed a test dataset comprising 322 questions. Each question was formatted as either fully uppercase, fully lowercase, or a combination of upper and lower cases, ensuring that the dataset covered various ways diseases could be mentioned. Each entry in the dataset had a label (referred to as "node hit"), which corresponds to the respective disease SPOKE node.

Following are some examples of this data:

Example 1:

CHERUBISM ASSOCIATES GENE SH3BP2 ; Node hit = cherubism

Example 2:

noonan syndrome associates gene kras ; Node hit = Noonan syndrome

Example 3:

Neurofibromatosis 1 associates Gene NF1 ; Node hit = neurofibromatosis 1

We utilized the disease entity recognition module to extract and find the matching SPOKE disease nodes, subsequently comparing them with their respective labels (node hits). Finally, we calculated the retrieval accuracy by dividing the total number of questions from which disease nodes were correctly retrieved by the total number of questions. We got a retrieval accuracy of 99.7% using this method.

**Disease entity extraction - Comparative analysis**

We conducted an experiment on named entity recognition (NER) for extracting disease entities from the user query using three methods: (1) zero-shot prediction using GPT-3.5 (2) the well-known SciSpacy NLP model 'en_core_sci_sm' (https://allenai.github.io/scispacy/) and (3) a BERT model specifically trained for NER task in biomedicine, called 'biomed-ner-all' (<https://huggingface.co/d4data/biomedical-ner-all>). This experiment aims to evaluate the NER capabilities of zero-shot GPT models against the other two benchmark models, focusing on precision and recall metrics. We chose these metrics for specific reasons:

1. Precision: This metric reveals the model's ability to accurately identify True Positives while minimizing False Positives. It indicates how effectively the method filters out noise in the entity detection process.
2. Recall: This measure shows how well the model identifies all relevant True Positives without missing any (avoiding False Negatives). It demonstrates the model's effectiveness in extracting pertinent entities from user queries.

The optimal approach should achieve high scores in both precision and recall, indicating it can extract all crucial entities while avoiding irrelevant information in context retrieval.

For this experiment, we used the same test dataset employed for the retrieval analysis, which consists of 322 biomedical queries. Fig S1 shows the result of the experiment and Table A shows the exact values of the metrics.

The average runtime per text for GPT-3.5 is 0.58 seconds, while the average latency for the other two models is significantly lower, at 0.02 seconds for BioMed NER and 0.01 seconds for SciSpaCy. This comparison based on runtime is not entirely fair, as the GPT models are API-based, whereas the other two models are deployed locally on the server where the experiment is conducted. Therefore, the difference in latency is expected.

Based on the experiment, we concluded that although the zero-shot prediction using the GPT-3.5 method has higher latency, its significantly superior performance in NER, as demonstrated by precision and recall metrics, makes it the preferred choice. These metrics are essential because they ensure minimal noise and high performance in context retrieval. Additionally, the GPT based approach offers the advantage of flexibility, allowing the NER task to be extended to different entity types through prompt engineering (such as using few-shot exemplars, refer to the “KG-RAG extended version – A proof of concept” and the system prompt supplementary sections). In contrast, other methods are less adaptable since they are trained on specific labels for token classification. For these reasons, we decided to adopt the zero-shot prediction method using GPT-3.5 for the disease entity extraction process.


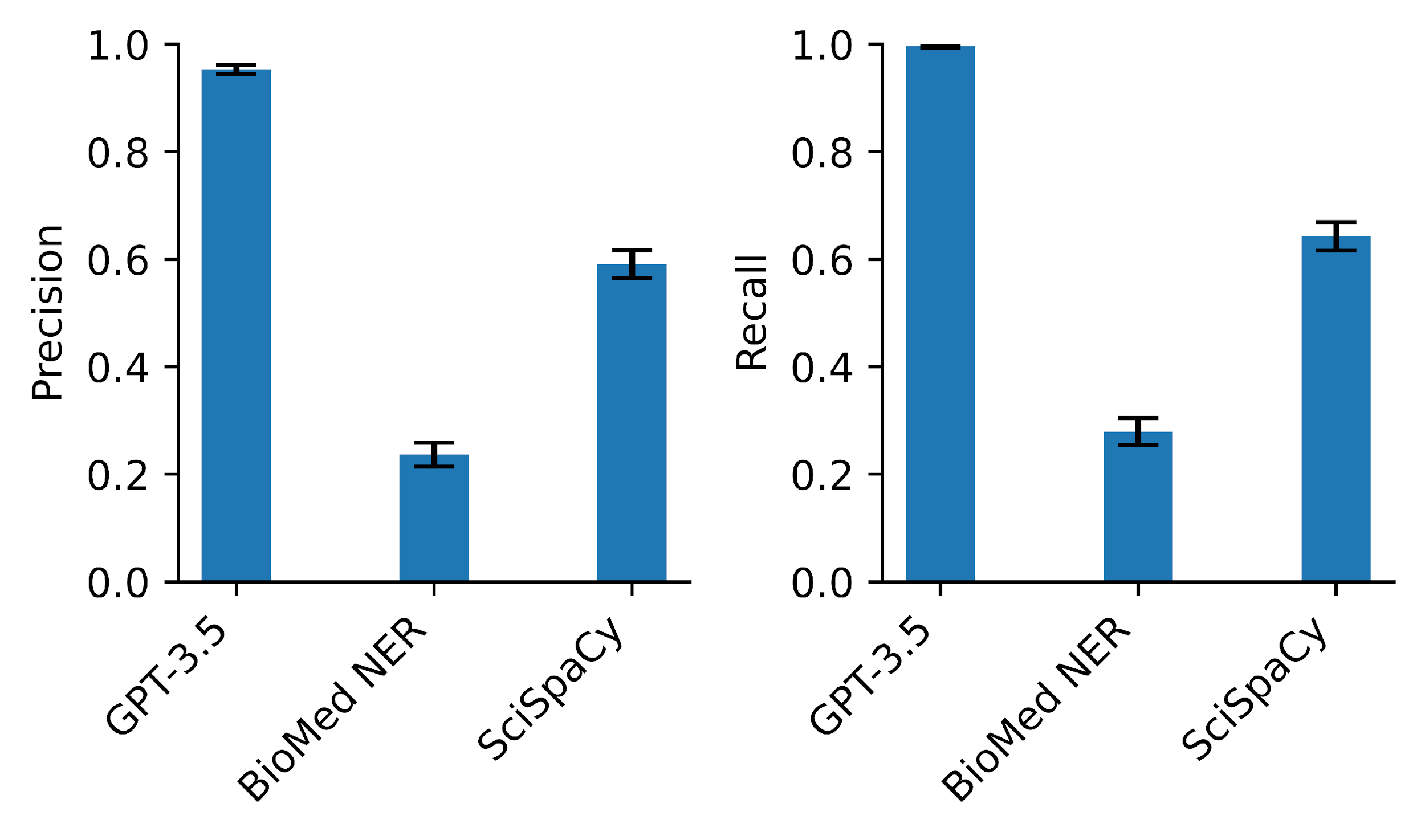


**Fig S1:** Bar plots that show the precision (left panel) and the recall (right panel) metrics for GPT-3.5 (zero-shot prediction), BioMed NER and SciSpaCy models in Named Entity Recognition (NER) task. Height of the bar shows the mean and the error bar indicates the sem (N=322).

**Table A:** Disease entity extraction comparison (mean±sem)

|  | **GPT-3.5 (zero-shot)** | **BioMed NER** | **SciSpaCy** |
| --- | --- | --- | --- |
| **Precision** | 0.95±0.01 | 0.24±0.02 | 0.6±0.03 |
| **Recall** | 0.996±0.003 | 0.28±0.03 | 0.64±0.03 |

**Validation dataset**

For the hyperparameter analysis, we generated two sets of validation data: prompts with single and two disease entities. Prompts with single disease entity featured questions about compounds treating ‘Disease X’ and diseases resembling ‘Disease X’. The first neighboring Compound and Disease nodes of ‘Disease X’ in SPOKE Knowledge Graph (KG) served as the ground truth for these prompts. For fetching the ground truth for Compounds that treat ‘Disease X’, we only considered those treatment associations that had a clinical phase >= 3. Prompts with two disease entities were more challenging since they involved questions about instances of a common node type associated with both 'Disease X' and 'Disease Y' leading to a graph with more hops. The ground truth for these prompts was obtained by running Cypher queries against SPOKE KG. Following are examples of these types of prompts along with the respective ground truths.

Example prompt with single disease entity:

Text : What compounds treat 'Alagille syndrome' and what diseases resemble 'Alagille syndrome'?

Compound Ground Truth : ['Odevixibat', 'Maralixibat']

Disease Ground Truth : ['optic disk drusen', 'intrahepatic cholestasis', 'Moyamoya disease', 'Williams-Beuren syndrome', 'pulmonary valve stenosis', 'xanthomatosis']

Example prompt with two disease entities:

Text : What are the Anatomy that are commonly associated with both tongue disease and Crouzon syndrome?

Ground Truth : ['mandible', 'foramen magnum', 'lower jaw region', 'face', 'masticatory muscle']

**Test dataset**

(a) DisGeNET

DisGeNET served as the data source that consolidated data about genes and genetic variants linked to human diseases [(Piñero et al. 2016)](https://paperpile.com/c/4FUUnF/t4Axg). We downloaded DisGeNET sqlite database and parsed “diseaseAttributes”, “geneAttributes”, “variantAttributes”, “geneDiseaseNetwork” and “variantDiseaseNetwork” tables to fetch Disease-Gene and Disease-Variant associations. Each association was annotated with PubMed ID, sentence from the PubMed article and an association score (a value between 0 and 1). For Disease-Gene associations, we used only those associations that had a score of 1. For Disease-Variant associations, we used only those associations whose score was > 0.9. This way we made sure to include only high quality associations for creating the test data. These associations were further used to create True/False statements. Following are two examples for such statements:

Example 1:

Text : Juvenile polyposis syndrome associates Gene SMAD4

Label : True

Example 2:

Text : Ulnar-mammary syndrome is not associated with Gene TBX3

Label : False

(b) MONDO

MONDO provided information about the ontological classification of Disease entities [(Vasilevsky et al. 2022)](https://paperpile.com/c/4FUUnF/WSKZu). We downloaded MONDO data available in Open Biomedical Ontologies (OBO) format. We parsed and processed those ontological associations without the “obsolete” flag set to True. An association is said to be ontological, if the entities were connected using the predicate “is_a”. These parsed data were further used to create True/False statements related to Disease classification. Following are two examples for such statements:

Example 1:

Text : cystic fibrosis is a respiratory system disorder

Label : True

Example 2:

Text : Disease ontology identifier for congenital generalized lipodystrophy type 2 is DOID:10588

Label : False

(c) SemMedDB

SemMedDB is a knowledge resource containing semantic predications (i.e. triplets representing subjects, predicates, and objects) extracted from all PubMed citations [(Kilicoglu et al. 2012)](https://paperpile.com/c/4FUUnF/1enR5). It can aid in hypothesis generation and literature-based discovery in the field of biomedicine. In this study, we utilized this resource to get the semantic predications on “what drug treats a disease”. To access SemMedDB, we utilized the API provided by the National Center for Advancing Translational Sciences (NCATS) Biomedical Data Translator project [(Fecho et al. 2022)](https://paperpile.com/c/4FUUnF/4cbnj). The API (<https://biothings.ncats.io/semmeddb/query>) allowed us to access the semantic predications and we included those with “TREATS” predicate. From the resulting predications, we included only those whose Subject entity belonged to one of the following types:

(i) Antibiotic

(ii) Pharmacologic Substance

(iii) Organic Chemical

(iv) Clinical Drug

(v) Inorganic Chemical

Each predication was also annotated with a count of PubMed articles supporting it. Hence, in addition to the above criteria, we included only those predications that had > 25 PubMed articles supporting it. The resulting data was used to create statements related to drugs and diseases. Following are two examples for such statements:

Example 1:

Text : metronidazole treats crohn's disease

Label : True

Example 2:

Text : melphalan treats melanoma

Label : True

(d) Monarch initiative and ROBOKOP

Monarch Initiative represents a collaborative and open science initiative with the goal of semantically unifying genotype-phenotype data from various species and sources [(Mungall et al. 2017)](https://paperpile.com/c/4FUUnF/jrp5Q). We used the BioLink API of Monarch initiative to fetch disease-gene and disease-variant associations (<https://api.monarchinitiative.org/api>). We fetched gene (and variant) nodes that were shared by disease pairs and this formed a graph involving two disease nodes. Along with the correct gene (and variant node) for a disease pair, we also randomly sampled four negative gene (and variant) samples. Next, a multiple-choice question was generated, comprising five choices with a single correct answer. We ensured that the order of options within each question was randomized, preventing any consistent placement of the correct answer among the choices across different questions.

ROBOKOP, which stands for Reasoning Over Biomedical Objects linked in Knowledge-Oriented Pathways, is a Knowledge Graph (KG) that was initially built to support the open biomedical question-answering application [(Bizon et al. 2019)](https://paperpile.com/c/4FUUnF/0eV5N). We used this KG to extract additional information on disease-variant associations. These associations were obtained by running Cypher queries on the ROBOKOP Neo4j browser and exporting the file from the browser in CSV format (<http://robokopkg.renci.org/browser/>). Similar to Monarch, we fetched variant nodes that were shared by disease pairs. Along with the correct variant for a disease pair, we also randomly sampled four negative variant samples. Next, a multiple-choice question was generated, comprising five choices with a single correct answer. We ensured that the order of options within each question was randomized, preventing any consistent placement of the correct answer among the choices across different questions.

Following are two examples of created MCQ:

Example 1:

Text : Out of the given list, which Gene is associated with psoriasis and Takayasu's arteritis. Given list is: SHTN1, HLA-B, SLC14A2, BTBD9, DTNB

Correct answer : HLA-B

Example 2:

Text : Out of the given list, which Variant is associated with atrophic gastritis and duodenal ulcer. Given list is: rs4459895, rs12203592, rs9275260, rs12524487, rs2294008

Correct answer : rs2294008

(e) RAG comparison test dataset

Objective of creating test dataset for RAG comparison is to test the context retrieval capability of KG-RAG and Cypher-RAG frameworks from SPOKE KG. Hence, we considered 100 random disease nodes in SPOKE KG. Next, for these 100 diseases we selected the corresponding Gene node that had the strongest association with it (i.e. the Gene with the least p-value from GWAS catalogue).

Next, we had two templates for creating the test question:

Template 1:

Is <entity 1> associated with <entity 2>?

Template 2:

What is the GWAS p-value for the association between <entity 1> and <entity 2>?

For every disease node, we chose one template at random. Subsequently, either the disease name or the gene name was inserted into the placeholders <entity 1> and <entity 2> with an equal probability. This step was taken to ensure that the queries traverse both directions within the graph, from disease to gene and from gene to disease.

To create the perturbed dataset, we followed the same methodology as above, except we converted the Disease and Gene names to lowercase. This was done to check the robustness in the context retrieval ability of KG-RAG and Cypher-RAG frameworks, irrespective of how the prompt was formulated.

Example 1:

Is hypertrophic cardiomyopathy associated with AMBRA1?

Example 2:

Is MARK3 associated with otosclerosis?

Example 3:

What is the GWAS p-value for the association between inflammatory bowel disease and SH2B3?

Example 4 (Perturbed prompt):

Is anxiety disorder associated with ntrk2?

True/False and MCQ questions/answers created using datasets mentioned in (a)-(d) were further reviewed by domain experts to remove any false positives.

**Cypher-RAG vs KG-RAG**

We compared the performances of Cypher-RAG and KG-RAG frameworks by utilizing the benchmark datasets such as True/False and MCQ datasets. The primary aim of this analysis was to evaluate the context retrieval capabilities of these frameworks in answering these questions. Our comparison was restricted to the GPT-4 model, excluding Llama2-13b and GPT-3.5-Turbo models, due to token limit considerations. Cypher-RAG consistently required tokens that approached the upper limit of the token size for Llama2-13b and GPT-3.5-Turbo (4096 tokens) and could potentially exceed this limit when processing the benchmark questions. This made GPT-4, with its higher 8192 token limit, the most suitable option for ensuring consistent and comprehensive comparisons across all questions in our analysis.

The results of our analysis, utilizing the GPT-4 model, revealed significant performance differences between Cypher-RAG and KG-RAG for both the True/False and MCQ datasets, with KG-RAG demonstrating superior performance (Fig S2, Table B). This performance gap was particularly pronounced in the True/False dataset. A deeper examination of the True/False results indicated that Cypher-RAG was unable to address 72% of the questions, responding with "I'm sorry, but I don't have the information to answer that question," even when the required information was present in the graph. In contrast, KG-RAG successfully answered these questions by effectively extracting relevant context from the graph.

A specific example illustrates this difference: For the True/False question "Noonan Syndrome associates Gene SOS1", Cypher-RAG failed to provide an answer, while KG-RAG correctly responded by extracting the relevant information about the disease “Noonan Syndrome” and the gene “SOS1” from the SPOKE graph. This example highlights a limitation in Cypher-RAG's context extraction capabilities, even when the necessary information is available within the graph structure. Hence, our analysis underscores the efficacy of KG-RAG in leveraging graph information to answer user queries, particularly in scenarios where direct query-based approaches like Cypher-RAG encounter difficulties in context extraction. These findings suggest that KG-RAG offers a more robust solution for extracting and utilizing relevant information from knowledge graphs in addressing user queries.


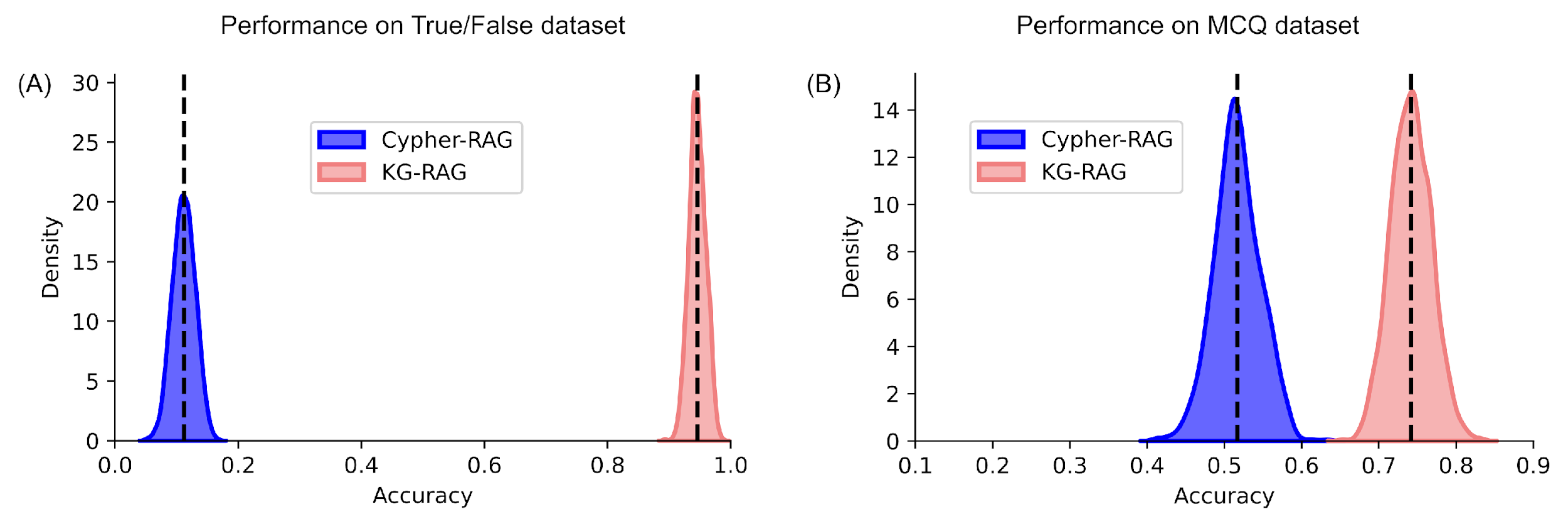
**Fig S2:** Comparative analysis of Cypher-RAG vs KG-RAG using (A) True/False and (B) MCQ benchmark datasets. x-axis shows the performance in terms of accuracy and y-axis shows the distribution density.

**Table B :** Comparative analysis between Cypher-RAG and KG-RAG using benchmark datasets (this was performed using GPT-4 model and values are mean±std)

| **True/False dataset** | | **MCQ dataset** | |
| --- | --- | --- | --- |
| **Cypher-RAG** | **KG-RAG** | **Cypher-RAG** | **KG-RAG** |
| 0.11±0.02 | 0.95±0.01 | 0.52±0.03 | 0.74±0.03 |

**KG-RAG extended version – A proof of concept**

KG-RAG approach is capable of handling multi-domain queries. As a proof of concept, we have separately indexed a broader set of 87,696 entities across various domains, including diseases, genes, proteins, enzymes, symptoms, compounds, reactions, anatomical structures, and side effects. This set represents a subset of our complete knowledge graph. Using this extended index, we ran several non-disease centric queries as shown below.

Example 1:

**Query:**

What protein does PAH gene encode?

**KG-RAG response:**

The PAH gene encodes the protein Phenylalanine-4-hydroxylase (PAH). [Provenance: UniProt]

Example 2:

**Query:**

What reactions do phenylalanine-4-hydroxylase catalyse?

**KG-RAG response:**

Phenylalanine-4-hydroxylase (PAH) catalyzes the following reactions:
 1. Reaction: L-phenylalanine + tetrahydrobiopterin + oxygen <=> dihydrobiopterin + L- tyrosine + H2O
 Provenance: KEGG database - https://www.genome.jp/kegg/

 2. Reaction: L-phenylalanine + tetrahydrobiopterin + oxygen <=> L-tyrosine + 4a- hydroxytetrahydrobiopterin
 Provenance: KEGG database - https://www.genome.jp/kegg/

 3. Reaction: L-phenylalanine + tetrahydropteridine + oxygen <=> L-tyrosine + 4a- hydroxy-5,6,7,8-tetrahydropteridine
 Provenance: KEGG database - https://www.genome.jp/kegg/

Example 3:

**Query:**

Does Balaglitazone downregulate any genes?

**KG-RAG response:**

Yes, Balaglitazone downregulates several genes. The genes that are downregulated by Balaglitazone include ADI1, ACOT9, IFRD2, CCNB1, H2BC12, RPS5, DHRS7, UGDH, KDELR2, WASHC4, LYPLA1, PRPF4, GOLT1B, HSPB1, AKR7A2, BAG3, GNAS, SOX4, DUSP6, CDC25B, FAT1, PRSS23, GRN, CANT1, HOXA10, IER3, PXN, CTNND1, BNIP3, HDAC6, KDM3A, SPDEF, ARID5B, ST6GALNAC2, DUSP4, TRAPPC6A, EPB41L2, IQGAP1, ASAH1, PHGDH, RGS2, FOXO4, TIMELESS, TCEA2, CD44, CDH3, DNAJA3, CDKN1B, PIK3R3, C2CD5, GLRX, HAT1, PTPRK, H2BC5, AKR7A2, ITFG1, KIFBP, S100A6, TRIM2, SLC2A6, ALDOC, ST3GAL5, NUCB2, PPARG, PTK2, H2BC12, APPBP2, ETV1, ERBB2, BNIP3L, TUBA1A, TGFB3, HMG20B, ALDH7A1, ACAT2, NIPAL3, ZNF33B, STMN1, DYNLT3, DNMT3A, EGF, CAST. [Provenance: CMAP/LINCS compound (trt_cp)]

These examples demonstrate the system's capability to handle more complex, multi-domain questions. Conducting a comprehensive multi-domain evaluation would be a valuable direction for future research, building upon the foundation established in this paper.

**System Prompts used**

1. Disease entity extraction

You are an expert disease entity extractor from a sentence and report it as JSON in the following format:

Diseases : <List of extracted entities>

Please report only Diseases. Do not report any other entities like Genes, Proteins, Enzymes etc.

1. Single disease entity validation

You are an expert biomedical researcher. For answering the Question at the end, you need to first read the Context provided. Then give your final answer by considering the context and your inherent knowledge on the topic. Give your answer in the following JSON format:

{Compounds:<list of compounds>, Diseases:<list of diseases>}

1. Two disease entity validation

You are an expert biomedical researcher. For answering the Question at the end, you need to first read the Context provided. Then give your final answer by considering the context and your inherent knowledge on the topic. Give your answer in the following JSON format:

{Nodes:<list of nodes>}

1. Prompt based text generation

You are an expert biomedical researcher. Answer the Question at the end with brevity.

1. KG-RAG based text generation

You are an expert biomedical researcher. For answering the Question at the end with brevity, you need to first read the Context provided. Then give your final answer briefly, by citing the Provenance information from the context. You can find Provenance from the Context statement 'Provenance of this association is <Provenance>'. Do not forget to cite the Provenance information. Note that, if Provenance is 'GWAS' report it as 'GWAS Catalog'. If Provenance is 'DISEASES' report it as 'DISEASES database - https://diseases.jensenlab.org'. Additionally, when providing drug or medication suggestions, give maximum information available and then advise the user to seek guidance from a healthcare professional as a precautionary measure.

1. True/False question

You are an expert biomedical researcher. For answering the Question at the end, you need to first read the Context provided. Based on that Context, provide your answer in the following JSON format.

{

"answer": "True"

}

OR

{

"answer": "False"

}

1. MCQ question

You are an expert biomedical researcher. For answering the Question at the end, you need to first read the Context provided. Based on that Context, provide your answer in the following JSON format for the Question asked.

{

"answer": <correct answer>

}

1. KG-RAG-Extended version (non-disease-centric questions): Biomedical entity extraction system prompt

You are an expert biomedical name entity extractor from a sentence. You are able to extract entities such as Diseases, Genes, Variants, Proteins, Symptoms, Side effects, Organisms, Pathways, Compounds, Reactions. Report your response as a JSON in the following format:

{"Entities": <List of extracted biomedical entities from the user input>}

IMPORTANT : Report the names mentioned in the user input, not the criteria they belong to.

Example 1:

User input : What are the proteins encoded by PINK1?

Response: {"Entities" : ["PINK1"]}

Example 2:

User input: Other than Parkinson's disease, what diseases do SNCA connect to?

Response: {"Entities": ["Parkinson's disease", "SNCA"]}

**Table C :** Prompts and responses using GPT-4 model - Prompt-based vs KG-RAG

| **Sl. no.** | **Prompt** | **GPT-4 (Prompt-based)** | **GPT-4 (KG-RAG)** |
| --- | --- | --- | --- |
| 1 | What are some protein markers associated with thoracic aortic aneurysm? | Some protein markers associated with thoracic aortic aneurysm include Matrix metalloproteinases (MMPs), C-reactive protein (CRP), and fibrillin-1. | The protein markers associated with thoracic aortic aneurysm include Chondroitin sulfate proteoglycan 4 (CSPG4), Matrix Gla protein (MGP), Interleukin-2 receptor subunit alpha (IL2RA), Interleukin-1 beta (IL1B), Myosin-10 (MYH10), Tropomyosin alpha-4 chain (TPM4), Tyrosine-protein kinase Mer (MERTK), and Stabilin-1 (STAB1). The provenance of these associations is the Cell Taxonomy database. |
| 2 | Are there any protein markers that show increased activity in adenocarcinoma? | Yes, certain protein markers like carcinoembryonic antigen (CEA), CA 19-9, and cytokeratins can show increased activity in adenocarcinoma. | Yes, there are several protein markers that show increased activity in adenocarcinoma. These include Keratin, type II cytoskeletal 7 (Cytokeratin-7) (CK-7) (Keratin-7) (K7) (Sarcolectin) (Type-II keratin Kb7), Anterior gradient protein 2 homolog (AG-2) (hAG-2) (HPC8) (Secreted cement gland protein XAG-2 homolog), Guanine deaminase (Guanase) (Guanine aminase) (3.5.4.3) (Guanine aminohydrolase) (GAH) (p51-nedasin), and Graves disease carrier protein (GDC) (Graves disease autoantigen) (GDA) (Mitochondrial solute carrier protein homolog) (Solute carrier family 25 member 16). The provenance of these associations is the Cell Taxonomy. |
| 3 | Do you know if ruxolitinib is approved as a pharmacologic treatment for vitiligo? | As of my knowledge up to date, ruxolitinib is not officially approved for the treatment of vitiligo. However, some clinical trials have shown promising results. Always consult with a healthcare provider for treatment options. | Yes, Ruxolitinib is associated with the treatment of vitiligo. This association is supported by data from ChEMBL and DrugCentral databases. However, it is always recommended to seek guidance from a healthcare professional for treatment options. (Provenance: ChEMBL, DrugCentral) |
| 4. | Are there any biomarkers that show increased profile in hydrocephalus? | Yes, certain biomarkers such as L1CAM, S100B, GFAP, and NSE have shown increased profiles in hydrocephalus. | Yes, the Transmembrane protein 119 (TMEM119) and P2Y purinoceptor 12 (P2RY12) show an increased profile in hydrocephalus. The provenance of this information is the Cell Taxonomy database. |
| 5. | Does drug dependence have any genetic factors? Do you have any statistical evidence from trustworthy sources for this? | Yes, drug dependence does have genetic factors. According to the National Institute on Drug Abuse, genetics account for about 40-60% of a person's vulnerability to drug addiction. | Yes, drug dependence does have genetic factors. The genes KAT2B and SLC25A16 have been associated with drug dependence. This information is backed by statistical evidence from the GWAS Catalog, with p-values of 4e-10 and 1e-09 respectively. |

**Table D :** Sources of test dataset used in this study

| True/False | | Multiple Choice Question (MCQ) | | RAG comparison | |
| --- | --- | --- | --- | --- | --- |
| Source(s) | Total questions | Source(s) | Total questions | Source(s) | Total questions |
| DisGeNET [(Piñero et al. 2016)](https://paperpile.com/c/4FUUnF/t4Axg), MONDO [(Vasilevsky et al. 2022)](https://paperpile.com/c/4FUUnF/WSKZu), SemMedDB [(Kilicoglu et al. 2012)](https://paperpile.com/c/4FUUnF/1enR5) | 311 | Monarch Initiative [(Mungall et al. 2017)](https://paperpile.com/c/4FUUnF/jrp5Q), ROBOKOP [(Bizon et al. 2019)](https://paperpile.com/c/4FUUnF/0eV5N) | 306 | SPOKE [(Morris et al. 2023)](https://paperpile.com/c/4FUUnF/gNIhV) | 100 |

**References**

[Bizon, Chris, Steven Cox, James Balhoff, Yaphet Kebede, Patrick Wang, Kenneth Morton, Karamarie Fecho, and Alexander Tropsha. 2019. “ROBOKOP KG and KGB: Integrated Knowledge Graphs from Federated Sources.” *Journal of Chemical Information and Modeling* 59 (12): 4968–73.](http://paperpile.com/b/4FUUnF/0eV5N)

[Deka, Pritam, Anna Jurek-Loughrey, and Deepak. 2022. “Improved Methods to Aid Unsupervised Evidence-Based Fact Checking for Online Heath News.” *Journal of Data Intelligence* 3 (4): 474–504.](http://paperpile.com/b/4FUUnF/5z4Xn)

[Fecho, Karamarie, Anne E. Thessen, Sergio E. Baranzini, Chris Bizon, Jennifer J. Hadlock, Sui Huang, Ryan T. Roper, et al. 2022. “Progress toward a Universal Biomedical Data Translator.” *Clinical and Translational Science* 15 (8): 1838–47.](http://paperpile.com/b/4FUUnF/4cbnj)

[Himmelstein, Daniel Scott, Antoine Lizee, Christine Hessler, Leo Brueggeman, Sabrina L. Chen, Dexter Hadley, Ari Green, Pouya Khankhanian, and Sergio E. Baranzini. 2017. “Systematic Integration of Biomedical Knowledge Prioritizes Drugs for Repurposing,” September. https://doi.org/](http://paperpile.com/b/4FUUnF/58J4)[10.7554/eLife.26726](http://dx.doi.org/10.7554/eLife.26726)[.](http://paperpile.com/b/4FUUnF/58J4)

[Kilicoglu, Halil, Dongwook Shin, Marcelo Fiszman, Graciela Rosemblat, and Thomas C. Rindflesch. 2012. “SemMedDB: A PubMed-Scale Repository of Biomedical Semantic Predications.” *Bioinformatics*  28 (23): 3158–60.](http://paperpile.com/b/4FUUnF/1enR5)

[Morris, John H., Karthik Soman, Rabia E. Akbas, Xiaoyuan Zhou, Brett Smith, Elaine C. Meng, Conrad C. Huang, et al. 2023. “The Scalable Precision Medicine Open Knowledge Engine (SPOKE): A Massive Knowledge Graph of Biomedical Information.” *Bioinformatics*  39 (2): btad080.](http://paperpile.com/b/4FUUnF/gNIhV)

[Mungall, Christopher J., Julie A. McMurry, Sebastian Köhler, James P. Balhoff, Charles Borromeo, Matthew Brush, Seth Carbon, et al. 2017. “The Monarch Initiative: An Integrative Data and Analytic Platform Connecting Phenotypes to Genotypes across Species.” *Nucleic Acids Research* 45 (D1): D712–22.](http://paperpile.com/b/4FUUnF/jrp5Q)

[Nelson, Charlotte A., Ana Uriarte Acuna, Amber M. Paul, Ryan T. Scott, Atul J. Butte, Egle Cekanaviciute, Sergio E. Baranzini, and Sylvain V. Costes. 2021. “Knowledge Network Embedding of Transcriptomic Data from Spaceflown Mice Uncovers Signs and Symptoms Associated with Terrestrial Diseases.” *Life*  11 (1). https://doi.org/](http://paperpile.com/b/4FUUnF/w8nG)[10.3390/life11010042](http://dx.doi.org/10.3390/life11010042)[.](http://paperpile.com/b/4FUUnF/w8nG)

[Nelson, Charlotte A., Riley Bove, Atul J. Butte, and Sergio E. Baranzini. 2021. “Embedding Electronic Health Records onto a Knowledge Network Recognizes Prodromal Features of Multiple Sclerosis and Predicts Diagnosis.” *Journal of the American Medical Informatics Association: JAMIA* 29 (3): 424–34.](http://paperpile.com/b/4FUUnF/LJbS)

[Piñero, Janet, Àlex Bravo, Núria Queralt-Rosinach, Alba Gutiérrez-Sacristán, Jordi Deu-Pons, Emilio Centeno, Javier García-García, Ferran Sanz, and Laura I. Furlong. 2016. “DisGeNET: A Comprehensive Platform Integrating Information on Human Disease-Associated Genes and Variants.” *Nucleic Acids Research* 45 (D1): D833–39.](http://paperpile.com/b/4FUUnF/t4Axg)

[Reimers, Nils, and Iryna Gurevych. 2019. “Sentence-BERT: Sentence Embeddings Using Siamese BERT-Networks.”](http://paperpile.com/b/4FUUnF/drEg) <http://arxiv.org/abs/1908.10084>[.](http://paperpile.com/b/4FUUnF/drEg)

[Soman, Karthik, Charlotte A. Nelson, Gabriel Cerono, Samuel M. Goldman, Sergio E. Baranzini, and Ethan G. Brown. 2023. “Early Detection of Parkinson’s Disease through Enriching the Electronic Health Record Using a Biomedical Knowledge Graph.” *Frontiers of Medicine* 10 (May): 1081087.](http://paperpile.com/b/4FUUnF/gqev)

[Tang, Alice S., Katherine P. Rankin, Gabriel Cerono, Silvia Miramontes, Hunter Mills, Jacquelyn Roger, Billy Zeng, et al. 2024. “Leveraging Electronic Health Records and Knowledge Networks for Alzheimer’s Disease Prediction and Sex-Specific Biological Insights.” *Nature Aging* 4 (3): 379–95.](http://paperpile.com/b/4FUUnF/zHl4)

[Vasilevsky, Nicole A., Nicolas A. Matentzoglu, Sabrina Toro, Joseph E. Flack, Harshad Hegde, Deepak R. Unni, Gioconda F. Alyea, et al. 2022. “Mondo: Unifying Diseases for the World, by the World.” *medRxiv*. https://doi.org/](http://paperpile.com/b/4FUUnF/WSKZu)[10.1101/2022.04.13.22273750](http://dx.doi.org/10.1101/2022.04.13.22273750)[.](http://paperpile.com/b/4FUUnF/WSKZu)
